# Evaluation of HER2 status and characteristics by next-generation sequencing in breast cancer

**DOI:** 10.3389/fmolb.2026.1873228

**Published:** 2026-06-23

**Authors:** Nanqiu Liu, Chongyang Ren, Yongqi Ren, Shuxuan Deng, Yan He, Xinze Lv, Zhou Zhang, Cheukfai Li, Li Cao, Kai Li, Hsiaopei Mok, Lingzhu Wen, Yulei Wang, Guochun Zhang, Ning Liao

**Affiliations:** 1 Department of Breast Surgery, Guangdong Provincial People’s Hospital, Guangdong Academy of Medical Sciences, Southern Medical University, Guangzhou, Guangdong, China; 2 Shantou University Medical College, Shantou, Guangdong, China; 3 Medical Marketing Department, Burning Rock Biotech, Guangzhou, Guangdong, China

**Keywords:** breast cancer, fluorescence *in situ* hybridization (FISH), human epidermal growth factor receptor 2 (HER2), immunohistochemistry (IHC), neoadjuvant therapy, next-generation sequencing (NGS)

## Abstract

**Background:**

Although next-generation sequencing (NGS) has been widely used to provide comprehensive somatic mutational information for individualized therapy in breast cancer patients, whether or not it could reliably evaluate HER2 status to guide targeted therapy when compared to the current IHC/FISH standard criteria still lacks sufficient validations.

**Methods:**

We analyzed the clinicopathological and NGS somatic genomic data of 1,171 Chinese breast cancer patients. A commercial 520 gene panel was used to generate genomic variation data by NGS in breast cancer samples. We compared the concordance of HER2 status between NGS evaluation and IHC/FISH criteria. We further defined the amplification status of HER2 based on the length of amplification region beginning from ERBB2 and extending to adjacent genes by focal (<1 megabase) versus broad (>1 megabase) amplification and stratified the cohort accordingly to investigate its potential in predicting efficacy of trastuzumab-based neoadjuvant therapy.

**Results:**

NGS showed high concordance with FISH/IHC-defined HER2 status with a sensitivity of 95.7%, specificity of 94.2%, and overall concordance rate of 94.9%. NGS characterized the genomic landscape of enrolled patients, identifying frequent mutations including TP53, ERBB2, PIK3CA, CDK12, MYC, and CCND1. The focal amplification subgroup exhibited significantly higher gene copy number (GCN) (31.7 vs 10.2, p < 0.001), greater HER2 positivity rates (98.3% vs. 78.3%, p < 0.001), higher IHC 3+ prevalence (91.7% vs. 48.5%, p < 0.001) and increased FISH + proportion (98.5% vs. 77.9%, p < 0.001). In patients receiving HER2-targeted neoadjuvant therapy, the focal amplification group had a higher pCR rate compared with broad amplification group (46.0% vs. 20.0%, p = 0.031).

**Conclusion:**

NGS demonstrates comparable capability to IHC/FISH in HER2 status interpretation and provides additional genomic information. Higher HER2 NGS copy numbers and focal amplification may be associated with a higher pCR rate.

## 1 Introduction

Human epidermal growth factor receptor 2 (HER2) positive breast cancer accounts for about 20% to 25% of all breast cancers (Siegel et al., 2022). Novel HER2-targeted therapies with mechanisms that do not rely on HER2 protein overexpression have recently been developed. Novel antibody-drug conjugates (ADCs), such as trastuzumab deruxtecan (T-DXd), has a response rate of 40% in metastatic breast cancer patients with low HER2 expression (IHC 1+ or 2+) (Modi et al., 2020; Tamura et al., 2019). Other novel treatments targeting the HER2 protein include HER2 vaccines and bispecific antibodies (Mittendorf et al., 2019; Meric-Bernstam et al., 2022; Weisser et al., 2023). Given these therapeutic advances, new methods to comprehensively evaluate HER2 status of patients are needed to screen for more patients who may benefit from these novel therapies.

At present, immunohistochemistry (IHC) and fluorescence *in situ* hybridization (FISH) are still the standard methods used to determine HER2 status based on guidelines published jointly by the American Society of Clinical Oncology (ASCO) and the College of American Pathologists (CAP) (Wolff et al., 2013; Wolff et al., 2018; Wolff et al., 2007). The 2018 version of the guidelines provide rather complex criteria that allow patients who would have previously been classified as equivocal HER2 to be classified as either HER2 positive or HER2 negative. However, this binary classification (as either HER2 positive or HER2 negative) does not allow the identification of patients with equivocal or low HER2 expression. This is particularly important because while aberrant HER2 signaling may play a much less dominant role in these patients, it is still targetable, and these patients may benefit from novel HER2-targeted treatment. The 2023 version of the guidelines acknowledge a new indication for T-DXd in breast cancers where HER2 is not overexpressed or amplified (Wolff et al., 2023), but shows IHC 1+ or 2+ without amplification by *in situ* hybridization, further suggesting it is necessary to utilize new approach to detecting HER2 status to gain additional information for helping formulate targeted-therapy regimens.

An ideal methodology to determine HER2 status should meet several performance criteria: 1) be compatible with the current binary classification system so that strongly HER2-positive patients can receive conventional anti-HER2 treatments; 2) provide quantitative information to identify patients expressing low levels of HER2 who could be candidates for novel HER2-targeted treatments; and 3) be capable of detecting sequence alterations in HER2 and other mutated genes that are actionable by anti-HER2 TKIs or other targeted therapies. Next-generation sequencing (NGS) is a readily available candidate that satisfies these requirements. At present, few validation studies have verified the concordance of NGS results with IHC and FISH results, and no NGS studies have defined a HER2 low-amplification status that could be used for selecting novel therapies (Ross et al., 2017; Pfarr et al., 2017).

We hypothesized that NGS could be used as an accurate methodology to determine the HER2 amplification status, along with other clinically important genetic information. To this end, we examined 1171 pre-treatment primary breast cancer samples and correlated the results with complete clinical and pathological data from these patients. We assessed the concordance of NGS results with ASCO/CAP standards in terms of HER2 status interpretation. We aimed to establish that NGS is a comprehensive method that can reliably identify patients with different HER2 expression levels and HER2 mutations who could be candidates for novel therapies.

## 2 Materials and methods

### 2.1 Patient selection

Patients diagnosed with breast cancer at Guangdong Provincial People’s Hospital were retrospectively enrolled in a prospective genomic profiling study. Participation involved voluntary submission of tumor tissue samples for next-generation sequencing (NGS), as outlined in prior publications (Zhang et al., 2019). The study population was not selected based on clinical or pathological characteristics; however, only individuals fulfilling all of the following inclusion criteria were incorporated into the final analysis: (1) Patients had not received any form of systemic therapy prior to sample collection; (2) Tumor specimens were obtained via core needle biopsy or surgical excision of the primary lesion and preserved as formalin-fixed, paraffin-embedded (FFPE) tissue; (3) Genomic profiling was conducted using a targeted NGS panel covering 520 cancer-related genes; (4) HER2 status was assessed by immunohistochemistry (IHC) and/or fluorescence *in situ* hybridization (FISH); (5) Complete clinical and pathological data were available, including estrogen receptor (ER), progesterone receptor (PR), and Ki-67 status.

Molecular subtypes were classified using IHC-based surrogate definitions according to the 2013 St. Gallen Consensus guidelines (Tang and Tse, 2016). Cases lacking any of the above criteria were excluded. All participants provided written informed consent for genomic testing via NGS. This study was conducted according to the ethical principles of the Declaration of Helsinki, with the study protocol reviewed and approved by Guangdong Provincial People’s Hospital Ethics Committee (permission number KY2025-836-01).

### 2.2 HER2 testing procedures

All breast tumor specimens were sectioned from FFPE tissue blocks and initially assessed for HER2 protein expression using IHC with the HercepTest assay (Dako/Agilent, Santa Clara, CA, USA). A score of 3+, defined by intense and complete membrane staining in more than 10% of tumor cells, was considered HER2-positive. Cases exhibiting an equivocal IHC score of 2+, or those requiring further evaluation based on patient or clinician request, underwent reflex testing using fluorescence *in situ* hybridization (FISH). FISH analysis was performed with the HER2 IQFISH pharmDx kit (Dako/Agilent, Santa Clara, CA, USA), targeting HER2 gene amplification. Amplification was defined as a HER2/CEP17 signal ratio ≥2.0 and/or an average HER2 copy number greater than 6.0 signals per nucleus. All HER2 assessments were interpreted independently by trained pathologists or certified laboratory personnel following the 2023 ASCO/CAP guidelines (Chen et al., 2020a) (Wolff et al., 2018).

### 2.3 Tissue DNA extraction and targeted sequencing

Genomic DNA was isolated from FFPE breast tumor samples with a minimum of 10% tumor cellularity using the QIAamp DNA FFPE Tissue Kit (Qiagen, Valencia, CA, USA), following the manufacturer’s protocol. DNA quantification was conducted using the Qubit dsDNA High Sensitivity Assay on a Qubit fluorometer (Life Technologies, Carlsbad, CA, USA). Targeted sequencing was carried out using a 520-gene panel (OncoScreen Plus, Burning Rock Biotech, Guangzhou, China), encompassing 1.64 Mb of genomic regions, including the full exonic regions of 312 genes and selected exons, introns, and promoter regions of the remaining 208 genes, as previously described (Chen et al., 2020a; Chen et al., 2020b). Briefly, extracted DNA was sheared acoustically into fragments of approximately 200–400 bp, followed by size selection, end-repair, adapter ligation, PCR amplification, and hybridization with custom capture probes. The resulting libraries were assessed for fragment size and concentration before sequencing on an Illumina NextSeq500 platform (Illumina, San Diego, CA, USA) using paired-end reads, achieving an average target coverage depth of 1000×. Sequencing data were analyzed using a proprietary bioinformatics pipeline developed by Burning Rock Biotech. Reads were aligned to the human reference genome (hg19) using Burrows-Wheeler Aligner (BWA, version 0.7.10). Local realignment and base quality recalibration were performed with GATK (version 3.2, Broad Institute), and small variants—including single nucleotide variants (SNVs) and insertions/deletions (Indels)—were identified using GATK and VarScan (version 2.4.3, Genome Institute, Washington University). Structural variants (SVs) were detected using Factera (version 1.4.3), and copy number variations (CNVs) were inferred from normalized coverage depth, with adjustments for GC content and probe hybridization bias. A CNV was called when the gene region exhibited statistically significant deviation (p < 0.005) from the control baseline, with a copy number ratio >0.5. Copy number status was classified as follows: amplification (≥2.75 copies), deletion (≤1.5 copies), and normal diploid (2 copies).

### 2.4 Determination of ERBB2 amplification region subtypes

To further characterize samples with potential ERBB2 amplification, subtype classification was performed based on focality analysis. Specifically, for each genomic segment, the Kullback-Leibler (KL) divergence was calculated between the copy number of the ERBB2 gene and that of its neighboring genes, including *NF1, RAD51D, CDK12, RARA, STAT3, BRCA1, SPOP, RNF43, RAD51C, BRIP1, CD79B, PRKAR1A, SOX9*, and *RPTOR*. A KL divergence score ≥8 was considered indicative of a significant copy number difference, suggesting independent amplification. A lower score implied the possibility of co-amplification across the region. The size of the contiguous amplified genomic region was then assessed, beginning from ERBB2 and extending to adjacent genes. If the length of this region was <1 megabase (Mb), the amplification was classified as focal. Regions larger than 1 Mb were designated as broad, representing candidate chromosomal-level amplifications. To further support classification into broad (polysomic) status, the estimated ERBB2 gene copy number (GCN) in tumor cells was evaluated. A GCN ≥5 was considered supportive of polysomy. The GCN was calculated using the formula:

$$\text{GCN}=\left(\text{absolute copy number}-2\right)/\text{ tumor purity}+2,$$

where tumor purity was estimated based on a combination of copy number variation and variant allele frequency (VAF) using a grid search optimization algorithm to identify the best-fit purity value.

### 2.5 Neoadjuvant therapy and treatment outcomes

Neoadjuvant systemic treatments were administered based on the patient’s HER2 and hormone receptor (HR) status, as well as physician discretion. Regimens included combinations of standard chemotherapeutic agents and HER2-targeted therapies, such as taxanes (T), epirubicin (E), doxorubicin (A), cyclophosphamide (C), 5-fluorouracil (F), carboplatin (Cb), capecitabine (X), trastuzumab (T), and pertuzumab (P). All drugs were delivered following established clinical dosing schedules. Treatment efficacy was evaluated by pathological complete response (pCR), assessed on both the surgically resected breast specimen and excised regional lymph nodes following completion of neoadjuvant therapy. pCR was defined as the absence of any residual invasive carcinoma both in the breast and lymph nodes, as determined by hematoxylin and eosin (H&E) staining.

### 2.6 Statistical analysis

All statistical analyses were conducted using the R statistical environment (version 4.0.5; R Foundation for Statistical Computing, Vienna, Austria). Descriptive statistics were applied to summarize clinical characteristics and genomic data. Comparative analyses of mutation profiles among different treatment response groups in our cohort were performed using either Fisher’s exact test or the Wilcoxon signed-rank test, depending on data type and distribution. Two-sided p-value < 0.05 was considered statistically significant.

## 3 Results

### 3.1 Baseline characteristics of the cohort

A total of 1,171 female patients with breast cancer were included in this study, with a median age of 49 years (range: 42–56 years). Most patients were diagnosed with early-stage disease (stage I or II), accounting for 64.5% (n = 756) of the cohort. Hormone receptor (HR)-positive tumors were predominant, observed in 68.7% (n = 804) of patients. Based on HER2 immunohistochemistry (IHC) and fluorescence *in situ* hybridization (FISH) results interpreted according to the 2018 ASCO/CAP guidelines, patients were stratified into three subgroups: HER2-zero: 149 patients (12.7%) had IHC 0 staining; HER2-low: 399 patients (34.1%) had IHC 1+ or 2+ with negative FISH results; HER2-positive: 560 patients (47.8%) had IHC 3+ or IHC 2+ with positive FISH results. In terms of HR and HER2 subtype distribution, HR-positive/HER2-negative cases comprised 39.2% (n = 459) of the population, while triple-negative breast cancer (TNBC) accounted for 6.7% (n = 78). Detailed clinicopathological characteristics of the entire cohort and individual subgroups are presented in Table 1.

**TABLE 1:** Clinicopathological characteristics of included patients.

Characteristics	Overall (n = 1171)
Age	
Mean (SD)	49.67 (10.71)
Median [IQR]	49.00 [42.00, 56.00]
Menopausal_status	
Post	513 (43.8)
Pre	606 (51.8)
Unknown	52 (4.4)
Tumor_stage	
T1-T2	944 (80.6)
T3-T4	88 (7.5)
Tis	30 (2.6)
Unknown	109 (9.3)
Lymph_node_stage	
N0-N1	865 (73.9)
N2-N3	236 (20.2)
Unknown	70 (6.0)
Metastasis_stage	
M0	1042 (89.0)
M1	67 (5.7)
Mx	61 (5.2)
MX	1 (0.1)
Pathologic_stage	
I-II	784 (67.0)
II-IV	299 (25.5)
Unknown	88 (7.5)
Histological_grade	
1	30 (2.6)
2	523 (44.7)
3	472 (40.3)
Unknown	146 (12.5)
Group	
HER2-zero	149 (12.7)
HER2-low	399 (34.1)
HER2-positive	560 (47.8)
Unknown	63 (5.4)
ER_status	
N	326 (27.8)
P	767 (65.5)
Unknown	78 (6.7)
PR_status	
N	406 (34.7)
P	685 (58.5)
Unknown	80 (6.8)
HR_status	
N	286 (24.4)
P	804 (68.7)
Unknown	81 (6.9)
HR_HER2_status	
HR-HER2+	208 (17.8)
HR + HER2-	459 (39.2)
HR + HER2+	343 (29.3)
TNBC	78 (6.7)
Unknown	83 (7.1)
Ki67_expression_status	
High	651 (55.6)
Low	441 (37.7)
Unknown	79 (6.7)

ER, Estrogen Receptor; PR, Progesterone Receptor; HR, Hormone Receptor; TNBC, Triple Negative Breast Cancer.

### 3.2 Comparison of ERBB2 Amplification/Overexpression detection between FISH, NGS, and IHC

**FIGURE 1:**
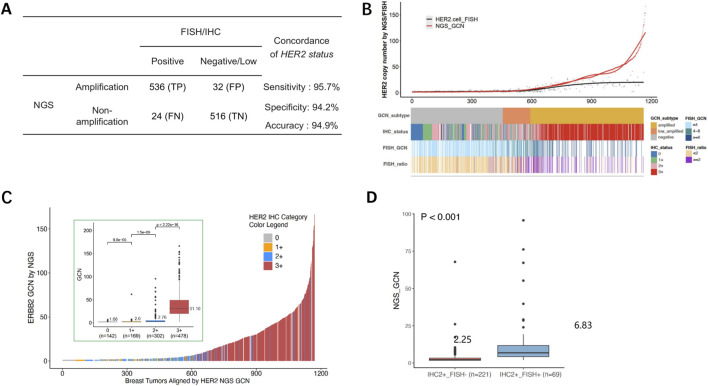
Comparison of ERBB2 Amplification/Overexpression Detection Between FISH, NGS, and IHC. HER2 status detected by NGS showed good concordance with IHC/FISH results **(A)**. A concordance was observed between the NGS GCN amplification and upgrade with FISH **(B)** and with IHC **(C)**. A distinct difference in gene copy number between IHC2+ FISH- and IHC2+ FISH+ **(D)**.

In a comparative analysis of 1,108 breast cancer cases, we assessed HER2/ERBB2 status using IHC, FISH, and NGS. ERBB2 amplification detected via NGS showed a high concordance with HER2 positivity defined by IHC and/or FISH, yielding a sensitivity of 95.7%, specificity of 94.2%, and an overall agreement rate of 94.9% (Figure 1A). These findings support the notion that NGS-based detection of gene amplification possesses comparable clinical significance to conventional protein and *in situ* hybridization methods, and further highlight the potential utility of NGS-derived quantitative metrics in clinical decision-making. To further examine concordance at the quantitative level, we analyzed the correlation between copy number values derived from NGS and the mean ERBB2 signals per nucleus obtained via FISH. A strong positive correlation was observed (R^2^ = 0.612, p < 0.001), though FISH-detected copy numbers demonstrated a plateau effect beyond a value of 25, suggesting limitations in resolution or interpretability at higher amplification levels using this method. We next categorized samples by amplification level using NGS (non-amplified, low-level, high-level), IHC scores (0, 1+, 2+, 3+), and FISH classification (GCN <4, 4–6, ≥6; HER2/CEP17 ratio <2 vs. ≥2). A consistent trend was observed across all three modalities, reinforcing the alignment between DNA-level amplification and protein expression patterns (Figure 1B). When stratified by IHC score, median ERBB2 copy numbers derived from NGS were 1.7 for IHC 0, 2.0 for IHC 1+, 2.8 for IHC 2+, and a markedly elevated 31.1 for IHC 3+. The pronounced increase from the IHC 2+ to 3+ group (p < 0.001) reflects a biologically significant threshold (Figure 1C), which likely contributes to the robust therapeutic response typically observed in IHC 3+ patients receiving HER2-targeted monoclonal antibodies. To better delineate the heterogeneity within the IHC 2+ population, we further subdivided this group into FISH-positive and FISH-negative cases. The ERBB2 copy number by NGS differed substantially between IHC 2+/FISH–and IHC 2+/FISH + subgroups (median 2.3 vs. 6.8, p < 0.001) (Figure 1D), providing indirect validation for the dual-testing approach used in clinical HER2 determination. These findings also suggest that quantitative NGS-based copy number analysis may offer a more refined and scalable biomarker to guide HER2-targeted therapy selection, especially in cases with borderline or equivocal IHC results.

### 3.3 Genomic and ERBB family mutation characteristics

**FIGURE 2:**
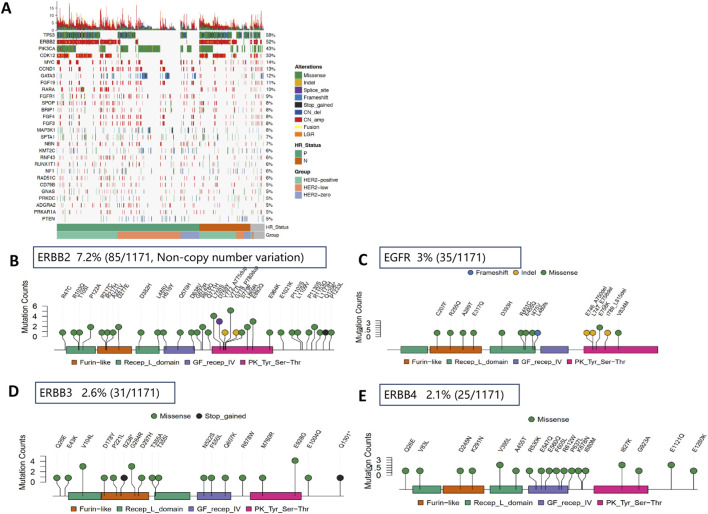
Genomic and ERBB Family Mutation Characteristic. Mutational landscape of included cohort **(A)** and high-frequency hotspot variants in ERBB2 **(B)**, EGFR **(D)**, ERBB3 **(D)** and ERBB4 **(E)**.

In this study, a total of 1,171 breast cancer samples from genomic testing were retrospectively analyzed to characterize the real-world mutational landscape in China. The most frequently mutated genes included TP53 (58%), ERBB2 (52%), and PIK3CA (43%). In addition, high-level gene amplifications were observed in CDK12 (33%), MYC (14%), and CCND1 (13%) (Figure 2A). Among these alterations, mutations in members of the ERBB family demonstrated potential clinical relevance.

Based on this large cohort, we further profiled the mutation characteristics of ERBB2, EGFR, ERBB3, and ERBB4 (Figures 2B–E). ERBB2 mutations were detected in approximately 7.2% of the cohort (85/1,171), with high-frequency hotspot variants such as V777L, L755S, D769Y, and E892Q—all located within the PTKc_HER2 kinase domain, suggesting potential functional and therapeutic implications. Mutation frequencies for EGFR, ERBB3, and ERBB4 were 3.0% (35/1,171), 2.6% (31/1,171), and 2.1% (25/1,171), respectively. Although no recurrent hotspot mutations were identified in these genes, notable alterations clustered within the PTKc_HER2 domain, including EGFR. E758K, ERBB3. E928G, and ERBB4. I827K, which may represent candidate activating mutations deserving further investigation.

To examine the interplay between ERBB2 amplification and concurrent mutations, we compared HER2 protein expression levels and ERBB2 copy numbers between the amplification-only group and the amplification-plus-mutation group. The results showed that the presence of co-occurring ERBB2 mutations did not significantly affect HER2 expression (Supplementary Figure S1). This indicates that although ERBB2 mutations may not influence HER2 protein levels, they could modulate downstream signaling activity and potentially impact sensitivity to HER2-targeted therapies—a hypothesis that warrants further functional and clinical validation.

### 3.4 Exploration of ERBB2 copy number characteristics

**FIGURE 3:**
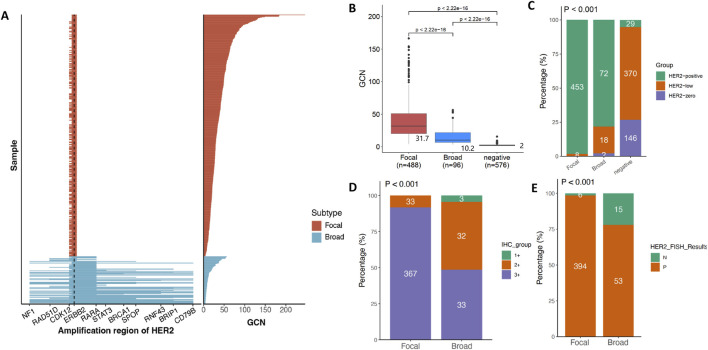
Unique amplification pattern of ERBB2. Patients were divided into ERBB2 focal amplification and broad amplification **(A)**. Focal amplification had a higher copy number **(B)**, HER2 positive rate **(C)**, IHC grade **(D)** and FISH amplification **(E)** compared with broad amplification.

Using a self-developed algorithm for interpreting ERBB2 amplification regions, we classified 584 breast cancer patients with ERBB2 amplification into two subtypes: Focal amplification (83.6%, 488/584) and Broad amplification (16.4%, 96/584) (Figure 3A). To investigate the biological and clinical distinctions between these two subtypes, we observed that the tumor ERBB2 copy number was significantly higher in the Focal group compared to the Broad group (median: 31.7 vs. 10.2, p < 0.001) (Figure 3B). Additionally, the proportion of HER2-positive expression (as determined by IHC and/or FISH) was markedly higher in the Focal group than in the Broad group (98.3% vs. 78.3%, p < 0.001) (Figure 3D). When stratified by IHC results, the Focal group comprised 91.7% (367/400) IHC 3+ cases and 8.3% (33/400) IHC 2+ cases, with no IHC 1+ cases. In contrast, the Broad group consisted of 48.5% (33/68) IHC 3+, 47.1% (32/68) IHC 2+, and 4.4% (3/68) IHC 1+ cases (Figure 3D). Similarly, based on FISH criteria, the Focal group had a significantly higher rate of HER2 amplification compared to the Broad group (98.5% vs. 77.9%, p < 0.001) (Figure 3E). These findings collectively suggest that Focal ERBB2 amplification is associated with a stronger oncogenic drive, reflected in both higher copy number levels and higher rates of HER2 overexpression. The data further imply that patients in the Focal amplification subgroup may derive greater benefit from anti-HER2 targeted therapies and could potentially be prioritized in clinical decision-making.

### 3.5 Molecular biomarkers of response to neoadjuvant therapy

A total of 130 patients with HER2-positive breast cancer received Trastuzumab-based treatment. The median age of the treatment cohort was 53 years. The majority of patients were in stage I–II, accounting for 57.7% (75/130). The proportions of hormone receptor-positive (HR+) and hormone receptor-negative (HR−) patients were comparable. Most patients exhibited high Ki67 expression (74.6%, 97/130). Among the cohort, 41.5% (54/130) achieved a pathological complete response (pCR) (Table 2). We compared the clinical characteristics between the pCR and non-pCR groups and found no statistically significant differences in baseline clinical features (Table 3). There was no significant difference in pCR rate between the single-target and dual-target treatment groups, nor between patients who received platinum-based chemotherapy and those who did not (Supplementary Figure S2).

**TABLE 2:** Clinicopathological characteristics of patients with Trastuzumab-based neoadjuvant therapy.

Characteristics	Trastuzumab-related drugs (n = 130)
Age	
Mean ± SD	50.52 ± 10.90
Median [IQR]	53.00 [43.00, 57.75]
Tumor_stage	
T1-T2	101 (77.7)
T3-T4	24 (18.5)
Tis	1 (0.8)
Unknown	4 (3.1)
Lymph_node_stage	
N0-N1	91 (70.0)
N2-N3	38 (29.2)
Unknown	1 (0.8)
Metastasis_stage	
M0	114 (87.7)
M1	15 (11.5)
Mx	1 (0.8)
Pathologic_stage	
I-II	75 (57.7)
III-IV	52 (40.0)
Unknown	3 (2.3)
Histological_grade	
1	1 (0.8)
2	61 (46.9)
3	57 (43.8)
Unknown	11 (8.5)
Group	
HER2-positive	127 (97.7)
Unknown	3 (2.3)
HR_status	
N	56 (43.1)
P	71 (54.6)
Unknown	3 (2.3)
Ki67.expression.status	
High	97 (74.6)
Low	33 (25.4)
NGS_GCN	
Mean (SD)	34.30 (26.58)
Median [IQR]	28.80 [15.84, 44.83]
pCR_status	
non-pCR	72 (55.4)
pCR	54 (41.5)
Unknown	4 (3.1)

pCR, pathological Complete Response.

**TABLE 3:** Comparison of patients with pCR and non-pCR.

Trastuzumab-related drugs	Non-pCR (n = 72)	pCR (n = 54)	p
Age			
Mean ± SD	51.79 ± 10.21	48.98 ± 11.61	0.152
Median [IQR]	53.00 [45.00, 58.00]	52.00 [40.00, 56.00]	
Menopausal_status			
Post	40 (55.6)	28 (51.9)	0.559
Pre	31 (43.1)	26 (48.1)	
Unknown	1 (1.4)	0 (0.0)	
Tumor_stage			
T1-T2	50 (69.4)	47 (87.0)	0.136
T3-T4	17 (23.6)	7 (13.0)	
Tis	1 (1.4)	0 (0.0)	
Unknown	4 (5.6)	0 (0.0)	
Lymph_node_stage			
N0-N1	48 (66.7)	39 (72.2)	0.695
N2-N3	23 (31.9)	15 (27.8)	
Unknown	1 (1.4)	0 (0.0)	
Metastasis_stage			
M0	63 (87.5)	47 (87.0)	0.788
M1	8 (11.1)	7 (13.0)	
Mx	1 (1.4)	0 (0.0)	
Pathologic_stage			
I-II	34 (47.2)	37 (68.5)	0.043
III-IV	35 (48.6)	17 (31.5)	
Unknown	3 (4.2)	0 (0.0)	
Histological_grade			
1	1 (1.4)	0 (0.0)	0.352
2	29 (40.3)	31 (57.4)	
3	32 (44.4)	23 (42.6)	
NA	10 (13.9)	0 (0.0)	
Group			
HER2-low	2 (2.8)	1 (1.9)	1.000
HER2-positive	70 (97.2)	53 (98.1)	
ER_status			
N	37 (51.4)	29 (53.7)	1.000
P	33 (45.8)	25 (46.3)	
Unknown	2 (2.8)	0 (0.0)	
PR_status			
N	40 (55.6)	34 (63.0)	0.376
P	30 (41.7)	20 (37.0)	
Unknown	2 (2.8)	0 (0.0)	
HR_status			
N	33 (45.8)	22 (40.7)	0.585
P	37 (51.4)	32 (59.3)	
Unknown	2 (2.8)	0 (0.0)	
Ki67_expression_status			
High	49 (68.1)	45 (83.3)	0.081
Low	23 (31.9)	9 (16.7)	
NGS_GCN			
Mean ± SD	33.16 ± 29.59	36.19 ± 23.00	0.533
Median [IQR]	24.73 [11.48, 47.10]	30.20 [23.66, 42.59]	

ER, Estrogen Receptor; PR, Progesterone Receptor; HR, Hormone Receptor; TNBC, Triple Negative Breast Cancer; pCR, pathological Complete Response.

**FIGURE 4:**
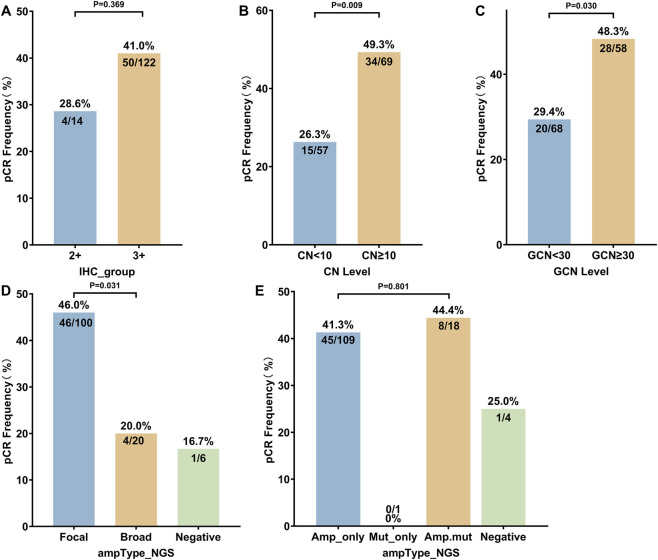
Correlation between pCR and HER2 amplification. There was no statistically significant difference between IHC grade and pCR **(A)**. Copy number **(B)**, gene copy number **(C)** and focal amplification **(D)** were associated with higher pCR rate. The co-presence of ERBB2 mutation did not affect the pCR **(E)**.

Further analysis was conducted to identify factors associated with pCR. Patients with HER2 IHC 3+ status had a higher pCR rate compared to those with IHC 2+ status (41.0% vs. 28.6%, p = 0.369) while there was no statistically significant difference (Figure 4A). When stratifying by copy number and genomic copy number with cutoffs of 10 and 30, respectively, patients in the high-copy group showed higher pCR rates than those in the low-copy group (49.3% vs. 35.1% for CN, p = 0.009, and 48.3% vs. 38.2% for GCN, p = 0.030) (Figures 4B,C), suggesting that high copy number may serve as a predictive marker for improved neoadjuvant efficacy. Notably, patients in the focal amplification group exhibited significantly higher pCR rates compared to the broad amplification (46.0% vs. 20.0%,p = 0.031) (Figure 4D), supporting the strong oncogenic drive of focal amplification and its potential role as a predictive biomarker for favorable neoadjuvant response. Meanwhile, the co-presence of ERBB2 mutation did not affect the pCR (Figure 4E).

**FIGURE 5:**
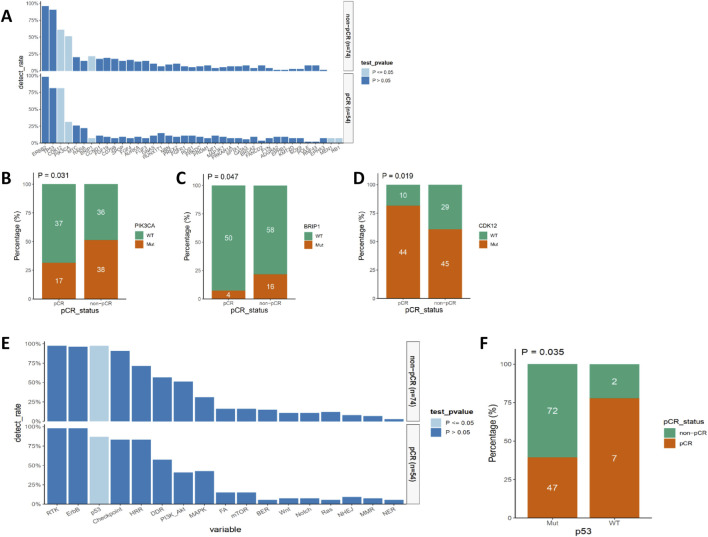
Correlation between neoadjuvant therapy outcomes and mutation characteristics. Mutational landscape between pCR and non-pCR group **(A)**. Mutations in the PIK3CA/BRIP genes were associated with a lower pCR rate **(B,C)**, while mutations in the CDK12 gene were correlated with a higher pCR rate **(D)**. Pathway analysis of above two groups **(E)** showed mutations in the p53 pathway had a poorer pCR rate **(F)**.

By comparing the mutational landscapes of the pCR and non-pCR groups (Figure 5A), we observed that PIK3CA and BRIP1 mutations were significantly less frequent in the pCR group (31.5% vs. 51.4%, p = 0.031; 7.4% vs. 21.5%, p = 0.047, respectively) (Figures 5B,C), whereas CDK12 mutations were more prevalent (81.5% vs. 60.8%, p = 0.019) (Figure 5D). Pathway-level analysis revealed that mutations in the p53 signaling pathway were associated with a lower pCR rate (39.5% vs. 77.8%, p = 0.035) (Figures 5E,F). These findings suggest that genomic profiling can aid in prognostic risk stratification and guide individualized treatment strategies.

## 4 Discussion

In current study, we evaluated the HER2 status of 1,171 patients using IHC/FISH and NGS respectively, and conducted a comparison. We identified that HER2 status interpreted by NGS showed good concordance with that detected by IHC/FISH. Moreover, NGS is able to provide a quantitative HER2 copy number for each breast cancer patient. The higher the HER2 copy number, the higher the corresponding IHC grade and FISH positivity probability, which is consistent with the trend observed in traditional testing methods. Previous study determined HER2 status via NGS and FISH in 57 patients, revealing strong concordance between two types of methods (Morsberger et al., 2022). Another small-scale study comparing NGS with conventional methods reported a sensitivity of 66.7%, specificity of 100%, and overall concordance of 93.5% in terms of HER2 status interpretation (Tay et al., 2024). Merlin et al. (Merlin et al., 2023) conducted an analysis using IHC and FISH on 31 breast cancer specimens while employing NGS for gene mutation selection, also identifying significant differences in ERBB2 CNV values among HER2-positive, HER2-low, and HER2-negative tumors. Nonetheless, the aforementioned studies have certain limitation due to their small sample sizes. Our study, based on a much larger cohort, validated the accuracy of NGS in construing HER2 status, suggesting its non-inferiority to conventional methods.

Our study revealed that NGS effectively stratified HER2-positive patients, predicted the efficacy of neoadjuvant therapy, and identified patients more likely to benefit from trastuzumab-based targeted therapy. Based our results, the HER2 gene exhibited two major amplification patterns: focal amplification and broad amplification. It was observed that the copy number of HER2 focal amplification was significantly higher than that of broad amplification. Patients with focal amplification were predominantly IHC 3+, FISH+, and HER2+, and presented a higher pCR rate compared with patients with broad amplification, indicating that even among patients classified as HER2-positive (i.e., FISH+), individual patients have different HER2 amplification patterns and varying CN levels, which may account for the discrepancy in responses to trastuzumab-based HER2-targeted therapy to some extent. Emerging evidence indicated that HER2-positive breast cancer responsiveness to targeted therapies was modulated by multiple factors including HER2 protein expression levels, HER2 gene copy number, and ER status (Shah et al., 2025; Zhang et al., 2012). We hypothesize that in patients with the focal amplification pattern, the tumorigenesis is primarily driven by HER2 amplification, and tumor growth relies heavily on the HER2 pathway, eliciting better response to trastuzumab-based targeted treatment. In contrast, tumor growth and metastasis in patients with broad amplification may not be solely associated with HER2, potentially resulting in the lower pCR rate after neoadjuvant therapy. The underlying mechanisms of above results warrant further investigation in future studies.

Moreover, our study highlighted that an advantage of NGS lied in its ability to detect additional mutations—such as those in ERBB2, PIK3CA, BRIP1, and CDK12—which may affect therapeutic response to trastuzumab. It’s reported that co-occurrence of activating ERBB2 mutations and ERBB2 amplification was associated with resistance to trastuzumab, pertuzumab, and first-generation TKIs (e.g., lapatinib) (Bose et al., 2013). Results from the plasmaMATCH trial (Turner et al., 2020) implied increasing ERBB2 mutation incidence in HER2-positive breast cancer with successive lines of HER2-targeted therapy. Preclinical and clinical studies indicated that irreversible TKIs (e.g., neratinib) exhibited activity against numerous ERBB2 point mutations. Findings from the SUMMIT basket trial (Jhaveri et al., 2023) revealed neratinib efficacy in metastatic ERBB2-mutant/ERBB2-non-amplified breast cancer, suggesting that NGS can be used to identify ERBB2-mutant breast cancer patients who deserved evaluation of TKI drugs, reducing the risk of targeted therapy resistance.

Additionally, previous research has established that PIK3CA mutations associated with poor anti-HER2 treatment responses (Jank et al., 2024; Kim et al., 2023), consistent with our results. Pathogenic PIK3CA mutations occur in approximately 23%-30% of HER2-positive breast cancers, where activation of the PI3K/AKT/mTOR pathway—triggered upon trastuzumab binding to HER2 domain IV or pertuzumab binding to domain II—served as a main mechanism driving resistance to HER2-targeted agents (Berns et al., 2007). We also found the BRIP1 gene, involving in DNA repair and chromosomal stability maintenance, was associated with poor pCR rate. BRIP1 gene belongs to the homologous recombination repair (HRR) pathway alongside BRCA1/2. BRIP1 mutation induces DNA damage repair deficiencies that promote oncogenesis, and its impact on anti-HER2 therapeutic efficacy merits further interrogation (Moyer et al., 2020).

Although NGS demonstrates good concordance with traditional IHC/FISH in interpreting HER2 status, caution is needed when applying NGS to ERBB2 detection. For HER2 evaluation, IHC/FISH remain standard methods. For equivocal or HER2-low cases, patients planned for neoadjuvant therapy, or those seeking comprehensive genomic guidance, NGS may be used as a complementary tool to provide copy number, amplification pattern, and concurrent mutations, especially in patients with poor response to standard neoadjuvant anti-HER2 therapy.

This study has several limitations. First, it is a single-center retrospective study and there are some biases in the included population. Multicenter prospective studies are required to further validate the accuracy of NGS in interpreting HER2 status. Second, the sample size of patient receiving neoadjuvant therapy was relatively insufficient. A larger sample size to further confirm the predictive potential of NGS in the context of neoadjuvant therapy is indispensable. Third, further research is needed to investigate the molecular mechanisms under different ERBB2 amplification patterns.

## 5 Conclusion

In summary, our study preliminarily validated the efficacy of NGS in interpreting HER2 status in breast cancer patients, demonstrating high concordance with conventional IHC/FISH. Higher HER2 copy numbers and focal amplification may be associated with a higher pCR rate. Future multicenter prospective studies are warranted to validate clinical interpretation criteria for NGS-based HER2 copy number and amplification patterns, and to evaluate their utility as predictive biomarkers for neoadjuvant therapy efficacy.

## Data Availability

The raw sequence data reported in this paper have been deposited in the Genome Sequence Archive (Genomics, Proteomics & Bioinformatics 2025) in National Genomics Data Center (Nucleic Acids Res 2025), China National Center for Bioinformation/ Beijing Institute of Genomics, Chinese Academy of Sciences (GSA-Human: HRA014010) that are publicly accessible at https://ngdc.cncb.ac.cn/gsa-human.
