# Supplementary material for: Evaluation of HER2 status and characteristics by next-generation sequencing in breast cancer
Source: Front Mol Biosci. 2026 Jun 23;13:1873228. doi: 10.3389/fmolb.2026.1873228 (PMC13337381; doi:10.3389/fmolb.2026.1873228)
Supplement: Supplementary file 1 [file DataSheet1.docx]

**
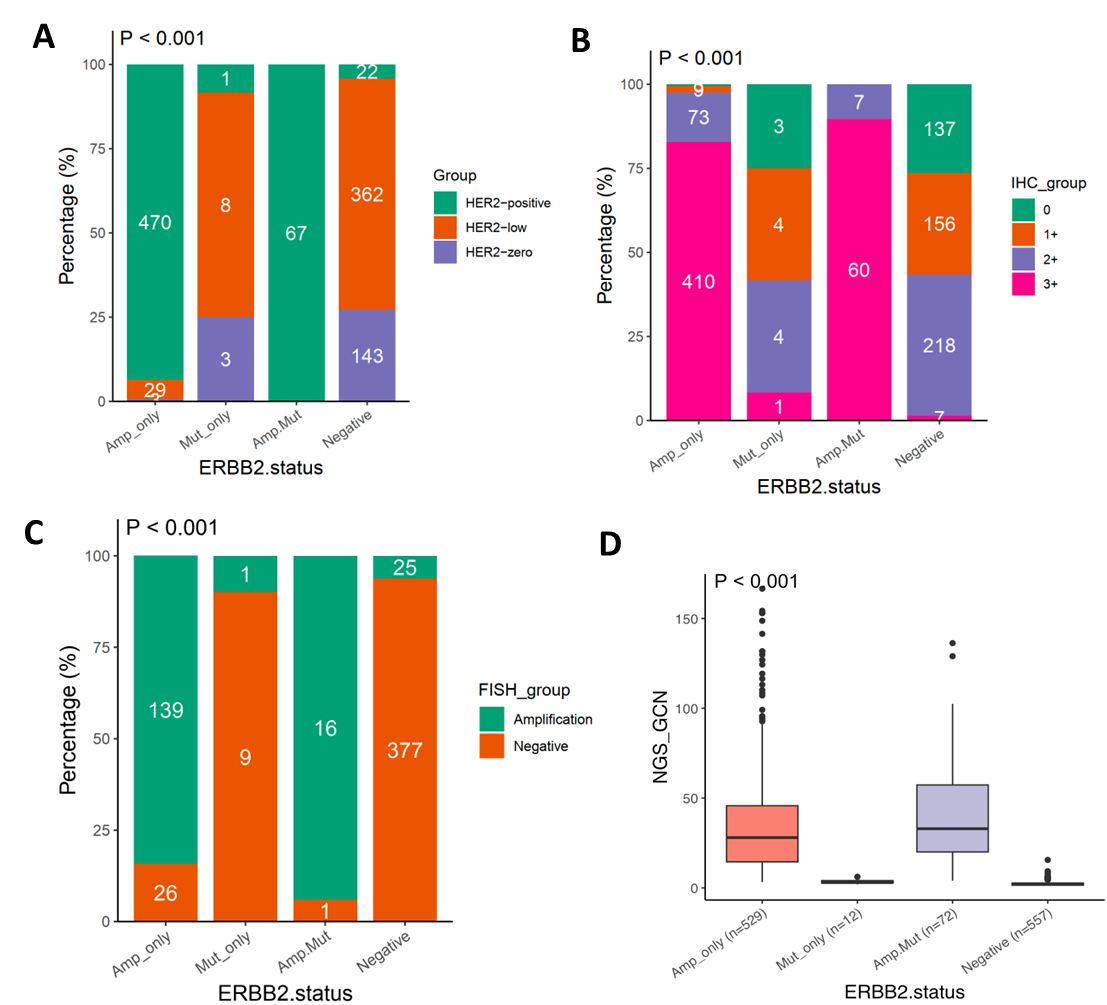
**

**Figure S1. Correlation between ERBB2 amplification and concurrent mutations.**

No significant differences were observed in the proportions of HER2 positive rate (A), IHC grade (B), FISH amplification (C), and NGS amplification (D) between the ERBB2 amplification with co-occurring mutation group and the ERBB2 amplification-only group.


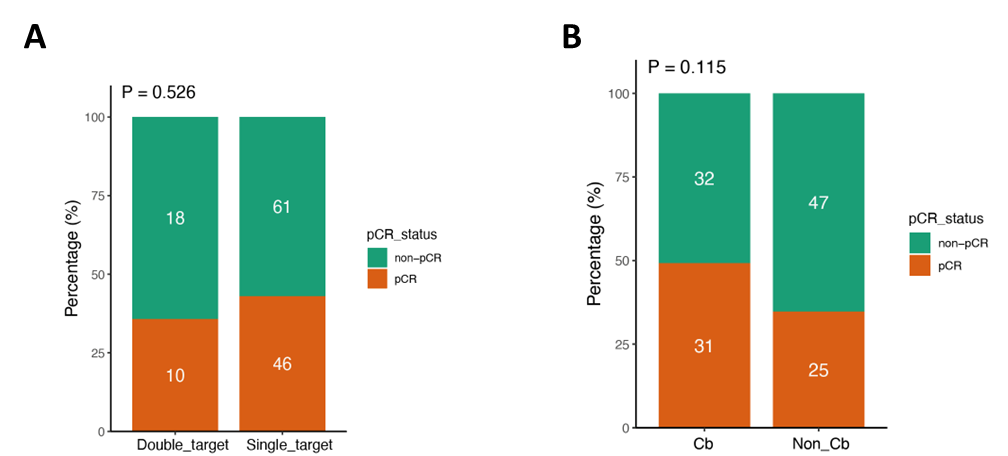


**Figure S2. Comparison of different neoadjuvant therapy regimens.** There’s no significant difference in pCR between patients with Trastuzumab and Trastuzumab plus Pertuzumab (A). The use of carboplatin-based agents in neoadjuvant therapy did not affect pCR (B). Cb: carboplatin.
